# Secondary parkinsonism associated with focal brain lesions

**DOI:** 10.3389/fneur.2024.1438885

**Published:** 2024-09-04

**Authors:** Rok Berlot, Anđela Pavlović, Maja Kojović

**Affiliations:** ^1^Department of Neurology, University Medical Centre Ljubljana, Ljubljana, Slovenia; ^2^Faculty of Medicine, University of Ljubljana, Ljubljana, Slovenia

**Keywords:** parkinsonism, lesion, secondary, reversible, MRI, diagnosis

## Abstract

Focal imaging abnormalities in patients with parkinsonism suggest secondary etiology and require a distinctive clinical approach to diagnosis and treatment. We review different entities presenting as secondary parkinsonism associated with structural brain lesions, with emphasis on the clinical course and neuroimaging findings. Secondary parkinsonism may be due to vascular causes, hydrocephalus, space-occupying lesions, metabolic causes (including acquired hepatocerebral degeneration, diabetic uremic encephalopathy, basal ganglia calcifications, osmotic demyelination syndrome), hypoxic-ischaemic brain injury, intoxications (including methanol, carbon monoxide, cyanide, carbon disulfide, manganese poisoning and illicit drugs), infections and immune causes. The onset can vary from acute to chronic. Both uni-and bilateral presentations are possible. Rigidity, bradykinesia and gait abnormalities are more common than rest tremor. Coexisting other movement disorders and additional associated neurological signs may point to the underlying diagnosis. Neuroimaging studies are an essential part in the diagnostic work-up of secondary parkinsonism and may point directly to the underlying etiology. We focus primarily on magnetic resonance imaging to illustrate how structural imaging combined with neurological assessment can lead to diagnosis. It is crucial that typical imaging abnormalities are recognized within the relevant clinical context. Many forms of secondary parkinsonism are reversible with elimination of the specific cause, while some may benefit from symptomatic treatment. This heterogeneous group of acquired disorders has also helped shape our knowledge of Parkinson’s disease and basal ganglia pathophysiology, while more recent findings in the field garner support for the network perspective on brain function and neurological disorders.

## 1 Introduction

Parkinsonism is a hypokinetic syndrome characterized by the core motor features that may present across a range of heterogenous disorders. It is defined by the presence of bradykinesia, accompanied by either rigidity, rest tremor or both (1). Secondary (acquired) parkinsonism (SP) implies that parkinsonism is due to an identifiable non-genetic and non-degenerative cause. The underlying etiology varies widely and may include medications, metabolic derangements, cerebrovascular causes, structural brain lesions, infections, immunological conditions and toxic influences (2). Although most patients with parkinsonism encountered in the clinical practice have idiopathic Parkinson’s disease (PD) or other neurodegenerative causes, SP is an important entity. From a historical perspective, SP has helped shape our knowledge of PD and gave insight into basal ganglia (BG) pathophysiology. From a clinician’s point of view, it is important that the course, prognosis and response to treatment of SP usually differ from PD and that many forms of SP are reversible with elimination of the specific cause.

SP related to metabolic and toxic causes presents with acute to subacute symptoms of symmetric bradykinesia, rigidity and prominent gait impairment, while rest tremor is minimal or absent. On the other hand, patients with space-occupying lesions or vascular causes usually manifest unilateral symptoms, which may even include rest tremor. A significant proportion of patients with SP will also manifest additional neurological, cognitive and psychiatric symptoms, depending on the lesion localization (2, 3).

In this review, we focus on the acquired forms of akinetic-rigid syndrome that are associated with distinct imaging findings. Consequently, we do not describe some other causes of SP, including iatrogenic causes which are the most common (2, 3). The focus on structural imaging, in particular magnetic resonance imaging (MRI), allows to illustrate how imaging techniques, combined with neurological assessment, can be utilized for early diagnosis that can lead to targeted interventions. Since neuroimaging is routinely applied in patients with parkinsonism, it is crucial for clinicians to recognize typical patterns of abnormalities within the relevant clinical context. For different etiologies, we emphasize the clinical course and treatment (summarized in Tables 1, 2), pathophysiological mechanism involved and characteristic imaging patterns that may lead to the diagnosis.

**Table 1:** Clinical features and treatment options of acquired parkinsonisms with focal lesions due to vascular etiology, structural damage and metabolic causes.

Etiology	Clinical context	Parkinsonism	Associated signs	Treatment	Levodopa response
Vascular					
Acute post-stroke parkinsonism	Sudden stable deficit or delayed occurrence after recovering from limb weakness	Usually unilateral, contralateral to the lesion	Pyramidal signs, hemisensory deficit	Control of vascular risk factors	Effective in isolated nigrostriatal damage, possible response in BG damage
Progressive vascular parkinsonism	Slowly progressive course in patients with white matter signal abnormalities on imaging	Symmetric with lower limb predominance and early postural instability	Cognitive impairment	Control of vascular risk factors	Rare response to high dose
Vascular malformations	Acute or subacute onset with slowly progressive or fluctuating course	Usually unilateral, contralateral to the malformation	Pyramidal signs, hemisensory deficit	Possibly reversible with embolisation treatment or surgery	Possible in rare cases of nigrostriatal damage
Hypoxic-ischaemic brain injury	Deterioration weeks or months after initial improvement	Symmetric, rigidity, bradykinesia, postural instability	Dystonia, bulbar signs, myoclonus, seizures	Symptomatic treatment of associated signs	Possible
Hydrocephalus					
Obstructive hydrocephalus	Usually years after shunt placement following shunt failure, sometimes paradoxical development as a response to shunting	Symmetric	Vertical gaze palsy, signs of increased ICP	Excellent response to shunting	Possible transient improvement in cases when shunt is not immediately effective
Idiopathic normal pressure hydrocephalus	Chronically progressive gait disturbance	Bradykinesia and rigidity common, lower limb predominance but may affect upper limbs	Gait apraxia, cognitive impairment, urinary incontinence	Ventriculo-peritoneal shunt	Variable
Space-occupying lesions					
Brain tumors	Usually subacutely progressive; possible delayed onset as a treatment complication	Usually unilateral	Focal neurological deficits, signs of hydrocephalus and/or increased ICP	Dexamethasone, surgical removal of tumor	Usually ineffective, possible effect in cases of midbrain tumors
Subdural haematoma	Subacute onset, often no history of trauma	Uni- or bilateral	Focal neurological deficits, confusion, signs of increased ICP	Surgical drainage	Usually ineffective
Metabolic causes					
Acquired hepatocerebral degeneration	Patients with chronic liver failure or porto-systemic shunt	Symmetric bradykinesia and rigidity, early gait disturbance and postural instability	Possible chorea, dystonia, ataxia and pyramidal signs	Supportive treatment, liver transplantation	Should be tested, variable response
Diabetic uremic encephalopathy	Patients with diabetes mellitus and end-stage renal disease, especially Asians	Acute onset	Impaired consciousness, bulbar signs, ataxia, seizures	Supportive treatment, adjusting blood glucose levels, hemodialysis	Uncertain
Hypocalcaemia and BG calcifications	Slowly progressive with associated signs in a younger patient with calcifications on imaging	Symmetric bradykinesia and rigidity, typical parkinsonian gait	Neuropsychiatric symptoms, additional movement disorders, symptoms of hypocalcaemia (paraesthesia, tetany, epileptic seizures)	May respond to normalization of serum calcium levels	Unresponsive
Osmotic demyelination syndrome	Deterioration after initial improvement in cases with rapid correction of hyponatremia	Usually symmetric, rarely asymmetric presentation or corticobasal syndrome	Pyramidal signs, pseudobulbar palsy	Supportive treatment	Possible in cases with midbrain demyelination

BG, basal ganglia; ICP, intracranial pressure.

**Table 2:** Clinical features and treatment options of acquired parkinsonisms with focal lesions due to toxic, infectious and immunological causes.

Etiology	Clinical context	Parkinsonism	Associated signs	Treatment	Levodopa resonse
Intoxications					
Methanol	Days after intoxication	Symmetric rigidity, bradyhypokinesia, hypomimia	Blindness, sometimes dystonia		Possible
Carbon monoxide	Delayed onset after recovery of consciousness, progressive course, stable disease or recovery	Acute onset, symmetric rigitidy, bradykinesia, sometimes rest tremor, hypomimia	Cognitive decline, dystonia	Possible spontaneous recovery with minimal residual symptoms	Ineffective
Cyanide	Recovery after attempted suicide or homicide	Acute or delayed onset	Dystonia, dysarthria		Possible in cases with SN degeneration
Carbon disulfide	Chronic exposure in industrial workers, treatment with disulfiram	Symmetric	Peripheral neuropathy, ataxia		Uncertain
Manganese	Chronic exposure in industrial workers, total parenteral nutrition, consumers of ephedrone	Symmetric with postural instability	Craniocervical and limb dystonia, »cock gait«		Should be tested, variable response
Infections					
Japanese B encephalitis	In most patients within the first month after regained consciousness	Symmetric bradykinesia, rigidity, hypomimia, possible tremor	Dystonia, pyramidal signs, cognitive impairment	Possible spontaneous recovery, often with residual deficits	Possible
*Mycoplasma pneumoniae*	After remission of respiratory symptoms, usually in children and young adults	Acute onset, symmetric	Dystonia, chorea, ataxia, dysarthria, neuropsychiatric symptoms	Usually spontaneous recovery, symptomatic treatment, occasionally immunosuppressive treatment	Uncertain
Neurocysticercosis	Rapid progression of atypical parkinsonism in a patient living in or traveling to an endemic area	Subacute onset, bilateral, may be asymmetric	Focal deficits, pyramidal signs, seizures, signs of increased ICP, neuropsychiatric symptoms	Antiparasitic drugs, sometimes steroid treatment or surgical excision	Uncertain
Opportunistic infections	Immunocompromised patient	Subacute onset	Focal signs, seizures, neuropsychiatric symptoms	Supportive and curative	Possible in cases with SN damage
Immunological					
Paraneoplastic	Resembling parkinsonism plus, often in younger individuals. First manifestation or later in disease course	Rapidly progressive, but can also be slower and mimic neurodegene-ration	Dystonia, gaze palsy, neuropsychiatric symptoms	Supportive, immunosuppressive and antineoplastic treatment	Possible partial improvement
Systemic autoimmune diseases	Usually in patients with an established autoimmune disease	Usually symmetric, but may be asymmetric	Focal signs, systemic symptoms	Immunosuppressive treatment	Ineffective
Multiple sclerosis	In patients with established multiple sclerosis, rarely newly diagnosed patients	Can be asymmetric	Focal signs	Steroids, immunomodulatory treatment	Possible in cases with damage to nigrostriatal fibers

BG, basal ganglia; ICP, intracranial pressure. SN, substantia nigra.

## 2 Methods

References for this review were identified by searching PubMed, references from individual identified articles, and literature recommended by co-authors and reviewers. PubMed was searched with the following terms: (Parkinson*) AND (focal lesion) AND (stroke or infarct or hemorrhage or hemorrhage or ischemia or vascular malformation or tumor or infection or inflammation or metabolic or toxic or toxic-metabolic or hydrocephalus). The search was unrestricted for dates. This process resulted in a reference list containing 483 articles, which included reviews, case reports or case series on SP with descriptions of focal abnormalities on computed tomography (CT) or MRI. Additional topics for the review as well as individual relevant papers were recommended by the reviewers. From this pool of articles, we curated the final reference list based on their relevance to the focus of this review and to provide a balanced overview of the various etiologies discussed in the manuscript. Where multiple articles were available on a single etiology, we prioritized those with larges case analyses, papers of historical significance, those describing MRI findings (except in cases where CT is more informative for the etiology) and with information about the quality of the response to dopaminergic therapy.

## 3 Causes of secondary parkinsonism

### 3.1 Vascular causes

#### 3.1.1 Acute post-stroke and progressive vascular parkinsonism

Vascular parkinsonism (VP) is caused by ischemic or, less commonly, hemorrhagic cerebrovascular insult. Parkinsonism is rare compared to other post-stroke movement disorders. In a cohort of 1,500 stroke patients, 56 patients exhibited movement disorders sequels, but only six of these had parkinsonism. Lesion localization included the BG in three cases and the frontal lobe, frontal and parietal lobe, and the brain stem in remaining cases (4).

There are two main types of VP. The first is acute post-stroke parkinsonism (APSP), which may be unilateral, or bilateral with unilateral predominance. The cause of infarction may be cardioembolic, atherothrombotic, lacunar or related to vasculitis, while hemorrhage is rare. APSP may present as sudden onset of parkinsonism with lack of progression or, more commonly, at the time the patient is recovering from limb weakness, typically within the first year after stroke (mean interval 4 months). This period, which is longer compared to other post-stroke movement disorders, may indicate the occurrence of secondary degeneration, i.e., trans-synaptic degeneration of substantia nigra (SN) with lesions involving the striatum (4, 5), or delayed functional alterations in distributed neuronal networks (4).

As expected, APSP may follow vascular damage in the contralateral midbrain (6, 7). In parkinsonism due to midbrain stroke, rest tremor is more common compared to other localizations. Additional focal neurological signs are usually observed, whereas pure hemiparkinsonism is rare (7, 8). Functional imaging confirms the involvement of dopaminergic pathways (9–13). Because striatal dopaminergic receptors are intact in the presence of presynaptic dopaminergic loss, symptomatic treatment with levodopa may be effective (8, 9, 11). There are occasional descriptions of patients with infarction or hemorrhage within the SN and ipsilateral hemiparkinsonism (6, 14). These paradoxical “wrong side” presentations remain unexplained, but could be related to the role of the ipsilateral striatum in the generation of rest tremor or could reflect the damage of crossed dopaminergic fibers from the substantia nigra to the thalamus, and are perhaps comparable to cases of PD with symptoms ipsilateral to the side of predominant dopaminergic nigrostriatal deficit (15–17).

APSP rarely results from stroke affecting the contralateral BG structures (18–20). Indeed, among 11 patients with striatal infarcts selected from 622 consecutive stroke patients, only one developed parkinsonism during the clinical follow-up (21). While it is rare for dopaminergic treatment to be effective in lesions outside the nigrostriatal pathway, there are descriptions of positive responses in individual cases with BG infarction (21–23). Damage to the nigrostriatal pathway can be identified with positron emission tomography (PET) and single photon emission computed tomography (SPECT) tracers. This can help narrow down patients that could benefit from dopaminergic treatment (13, 19).

Finally, APSP has also been described in lesions confined to the cortex. This could be a result of their more wide-spread impact on brain function (4, 24). For example, hemiparkinsonism in a patient with acute infarction of the contralateral anterior cingulate cortex could have resulted from network changes that lead to hypometabolism in functionally linked ipsilesional motor areas including the caudate, putamen, thalamus and SN (24). More commonly, however, subcortical structures are also affected and thus contribute to parkinsonism (25).

The second (“progressive”) form of VP has an insidious onset with a gradually progressive course and is associated with subcortical white matter (WM) T2/FLAIR hyperintensities and lacunar infarcts of the subcortical WM, BG and the brainstem (5, 26, 27). Parkinsonism predominates in the lower limbs and patients have short-stepping gait with early postural instability. This type of VP is somewhat controversial: there is no abnormal structural imaging pattern specific to this type of VP, and there is poor correlation between brain MRI hyperintensities and microangiopathic brain disease and parkinsonism from available clinicopathologic data (28). This may imply that a proportion of the patients labeled as VP may instead have PD or other neurodegenerative parkinsonism with co-existing leukoaraiosis, or an alternative diagnosis associated with WM changes, such as normal pressure hydrocephalus, CADASIL or rare forms of adult-onset leukodystrophy (28).

The term “cribriform state” or “Swiss Cheese Striatum” (SCS), refers to dilatations of the perivascular spaces leading to lacunar cysts in the BG (especially striatum) evident on MRI. Several bilateral or unilateral cases of parkinsonism have been reported in association with SCS (29, 30). However, no difference in the prevalence of parkinsonism or the presence of vascular risk factors was observed between patients with MRI evidence of SCS and matched controls (31). Thus, clinicians need to consider other causes before attributing parkinsonism to SCS.

#### 3.1.2 Vascular malformations

Vascular malformations (VM) are a rare but potentially reversible cause of SP. Symptoms may be due to the mass effect of the VM, parenchymal injury caused by ischemia or hemorrhage, or due to subtle metabolic alterations resulting from circulatory changes. For example, dural arteriovenous fistulae (DAVF) may cause parkinsonism by hypoperfusion of the BG and frontal WM or by impairing the drainage of the deep veins (32–35). The haemodynamic impairment caused by DAVF may also lead to clinical parkinsonism by accentuating dopaminergic deficiency in patients with preclinical PD. (36) MRI findings suggestive of DAVF are diffuse T2-weighted/FLAIR hyperintensities in the WM (that may also affect the BG) and tortuous flow voids of venous channels. A combination of parkinsonism and T1-weighted hyperintensities in the BG (related to venous congestion) is particularly suggestive of DAVF (37). Angiography remains the gold standard for diagnosis and provides detailed information for treatment planning. The extent of reversibility of symptoms with surgical removal or embolization treatment will depend on the mechanism involved.

### 3.2 Hydrocephalus

#### 3.2.1 Obstructive hydrocephalus

Parkinsonism is a rare complication of obstructive hydrocephalus (OH). If OH is caused by infratentorial malignant processes, it is difficult to conclude if symptoms arise from brainstem compression/infiltration or from secondary hydrocephalus (38). Nevertheless, cases of parkinsonism in non-neoplastic aqueductal stenosis confirm the causative role of increased cerebrospinal fluid (CSF) pressure (39, 40). Parkinsonism usually appears months or years after the initial presentation of OH, following repeated episodes of shunt failure. It is symmetric and may be associated with vertical gaze palsy (as part of Parinaud’s syndrome), thus resembling progressive supranuclear palsy (PSP) (41). It is likely a consequence of direct and immediately reversible compression of the BG, with excellent response to shunting. In cases where the shunt is not immediately effective, this may be due to predominant compression or torsion of nigrostriatal projections and dopaminergic replacement therapy may be effective (40, 42).

Clinicians should also be aware of paradoxical worsening of parkinsonism after shunting that occurs despite the normalization in the ventricular size. This is presumably caused by sudden alteration of the transtentorial pressure gradient or shunt overdrainage, which may damage nigrostriatal fibers (43, 44). In this scenario, the response to levodopa is good. Medications may be discontinued after a while, without recurrence of parkinsonism (45, 46).

#### 3.2.2 Idiopathic normal pressure hydrocephalus

Idiopathic normal pressure hydrocephalus (iNPH) presents with progressive dementia, gait apraxia and urinary incontinence, associated with ventriculomegaly and normal CSF pressure (47). There are no definitive pathological findings confirmatory of iNPH. In addition, ventricles enlarge with age, while gait abnormalities, incontinence and cognitive impairment are also common in elderly. Thus, when dealing with patients with parkinsonism and radiological findings of iNPH, clinicians should address the question if parkinsonism is a direct consequence of iNPH or the patient has another neurodegenerative disease that may present with enlarged ventricles (48). In iNPH, the lateral and third ventricles are enlarged out of proportion to the cortical sulcal enlargement, along with periventricular hypodensity on CT or high T2/FLAIR signal on MRI, which might represent transependymal exudate. Evans index is the most established measurement in the diagnosi of iNPH, and is defined as the ratio of the maximal distance between the lateral margins of the lateral ventricles to the inner skull diameter on the same transverse slice. The value of 0.3 or greater is typical for iNPH (49). Among other markers supportive of iNPH are increased anterior–posterior diameter of the lateral ventricles, acute callosal angle, upward bowing of the corpus callosum, and crowding of the sulci near the vertex accompanied by enlarged CSF spaces inferiorly, particularly in the Sylvian fissures (disproportionately enlarged subarachnoid space hydrocephalus, DESH) (50–52), Although characteristic gait impairment is the most well-known motor symptom of iNPH, bradykinesia and rigidity are relatively common, and may affect upper limbs (46). Some studies even reported greater involvement of upper vs. lower limbs, or exclusive upper limb parkinsonism (46). Gait disturbance and postural instability may improve with shunting. Even though less data is available on the response of the cardinal motor signs of parkinsonism, these may also improve in a proportion of patients after shunting (53). The response to levodopa is highly variable, from no effect or little improvement to excellent response (46). Nevertheless, levodopa responsiveness in iNPH is rare and suggests a primary neurodegenerative process of the nigrostriatal system, particularly when improvement is striking.

### 3.3 Space-occupying lesions

#### 3.3.1 Brain tumors

**Figure 1:**
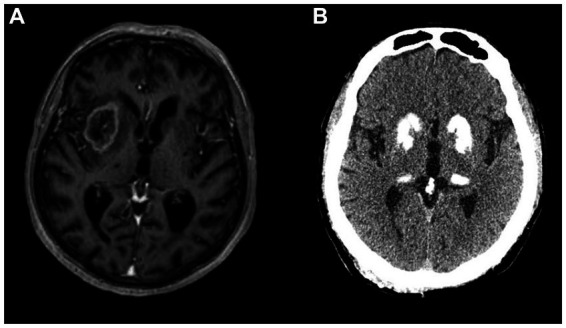
**(A)** Glioblastoma in right basal ganglia on gadolinium-enhanced T1-weighed MRI presenting with left hemiparkinsonism, gait instability and mild dysarthria. **(B)** Basal ganglia calcifications on CT scan in a patient with a disorder of calcium metabolism presenting with symmetric parkinsonism.

Brain tumors may lead to parkinsonism through several mechanisms, which may overlap. The most common is the pressure on the BG by the mass and its surrounding oedema, as in the cases of meningiomas and other supratentorial tumors not directly invading the BG (37, 54). The second is direct infiltration of the BG by glioma or lymphoma (Figure 1A) (55, 56). Next, brain tumors can cause SP by damaging the SN, through midbrain compression (e.g., craniopharyngeoma) or by infiltration (55, 57). Parkinsonism may also result from tumors involving the supplementary motor area or another strategic location that is disconnecting the pathways between the BG and the cortical motor areas (58–60). Finally, parkinsonism may arise in the setting of obstructive hydrocephalus caused by a tumor (38).

Clinical presentation and imaging findings depend on tumor localization, size, presence of oedema, and hydrocephalus. Parkinsonism is the presenting feature in small tumors involving the nigrostriatal pathway or individual BG structures. In contrast, larger tumors with surrounding oedema present with other neurological signs that may later be followd by parkinsonism. Hydrocephalus may be accompanied by signs of increased intracranial pressure, including headache, nausea, altered vision and decreased consciousness (61). Although cerebral tumors are not a common cause of parkinsonism, they should not be overlooked as a potentially curable cause. Parkinsonism is reversible when caused by compression rather than infiltration of the BG (58, 62, 63). Dopaminergic medications usually have no beneficial effect. However, distortion of the midbrain or infiltration resulting in SN cell loss may result in levodopa responsiveness (64, 65).

In addition to disease recurrence or progression, the appearance of parkinsonism in a patient with a history of brain tumor may indicate delayed treatment complication. Parkinsonism has been observed in children and adults after cranial radiotherapy. Pediatric cases have been associated with radiation induced globus pallidus (GP) lesions (66, 67). In adults, delayed parkinsonism not responsive to levodopa has been reported following whole brain irradiation, with MRI being consistent with leukoencephalopathy, likely caused by radiation-induced obliterating vasculopathy (68). Parkinsonism has also been observed in patients after systemic or intrathecal chemotherapy, in patients treated with immune checkpoint inhibitors for solid tumors and in oncological patients treated with amphotericin B (3, 69, 70).

#### 3.3.2 Subdural haematoma

Uni-and bilateral subdural haematomas (SDH) may rarely present with contralateral or bilateral parkinsonism, respectively (71–74). Symptoms may be caused by the mass effect of the SDH, resulting in mechanical compression of the BG or the nigrostriatal pathway, or by remote vascular disturbances and/or metabolic changes within the BG circuits (73). In addition, mechanical compression of the contralateral structures due to midline shift may result in ipsilesional parkinsonism (75). Parkinsonism develops subacutely, accompanied by headache, cognitive impairment and pyramidal signs. Quite often there is no history of head trauma, and symptoms appear to have developed spontaneously (as is often the case for chronic SDH in general), dictating a low threshold for requesting imaging in patients with subacute neurological deterioration. Surgical drainage of SDH usually leads to complete remission of the symptoms. Occasionally, in cases of bilateral SDH, remission is only partial (74).

### 3.4 Metabolic causes

#### 3.4.1 Acquired hepatocerebral degeneration

Acquired hepatocerebral degeneration (AHD) affects 1–2% of patients with chronic liver failure or porto-systemic shunt (76). Parkinsonism is its most common presentation. It is associated with early gait disturbance, falls and cognitive impairment. Chorea, orolingual dyskinesias, dystonia, ataxia and pyramidal tract signs may co-exist. The onset is usually insidious, the clinical course varies from stable to progressive (76). Symptoms of AHD arise from dysfunctional hepatic clearance and consequent accumulation of toxic substances in the brain. More specifically, parkinsonism arises from manganese accumulation in the BG with predilection for the pallidum. Accordingly, MRI shows bilateral pallidal T1 hyperintensities (77). High T1 signal may extend to the putamen, caudate and SN, but is only rarely seen in the cerebellum. Levodopa should be tried in every patient, but response is variable, from modest to dramatic, likely reflecting more than one mechanism of manganese-induced parkinsonism. While most patients have normal dopamine transporter uptake, consistent with postsynaptic parkinsonism, there are cases with reduced dopamine transporter uptake, suggesting that manganese accumulation may also cause degeneration of presynaptic dopaminergic nerve cells. Both parkinsonism and pathological MRI signal may improve after liver transplantation (78).

#### 3.4.2 Diabetic uremic encephalopathy

Acute bilateral BG lesions may appear in diabetic uremic patients. Patients with end-stage renal disease caused by long standing diabetes mellitus (particularly Asians) may develop acute parkinsonism, which may be accompanied by lethargy, bulbar impairment with dysarthria and dysphagia, ataxia, and seizures. MRI findings are striking, revealing bilateral oedema of the lentiform nuclei, which appears as hyperintense on T2/FLAIR, surrounded by a bright hyperintense rim resembling a fork – the so-called lentiform fork sign (79). MRI changes represent vasogenic oedema, caused by the breakdown of vascular autoregulation due to metabolic derangement. Improvement of parkinsonism with regression of imaging abnormalities is common after hemodialysis. This syndrome is distinct from the classical “uremic encephalopathy,” where cortical dysfunction manifests with seizures, cortical myoclonus and abnormal EEG.

#### 3.4.3 Parkinsonism due to hypocalcaemia and BG calcifications

Calcifications in the BG appear in 0.2–2% of routine CT scans, even more commonly in individuals over 50, and may be detected in almost 40% of 70-year-olds (80, 81). In the vast majority, such age-related calcifications are confined to the pallidum and have no clinical significance. When calcifications extend beyond the pallidum to involve the striatum, thalamus, dentate nuclei and cerebral WM, they are likely pathological (Figure 1B). They appear in primary familial BG calcification (formerly known as Fahr’s disease), a rare genetic neurodegenerative disorder (82). Further, pathological calcifications can occur in disorders of calcium metabolism, most commonly in hypoparathyroidism and pseudohypoparathyroidism (83). The recent literature review of 223 cases of BG calcifications due to hypoparathyroidism revealed parkinsonism as the most common movement disorder, followed by chorea, ataxia, tremor, dystonia or paroxysmal dyskinesias (84). These patients also had prominent neuropsychiatric symptoms, including cognitive decline, personality or behavioral changes, apathy, agitation, psychosis, depression and anxiety (84). The mean time between symptom onset and diagnosis is approximately a decade, suggesting very long diagnostic delay. In patients with hypoparathyroidism, parkinsonism most commonly does not respond to levodopa but may respond to restoration of calcium levels (84). In primary familial BG calcification, levodopa response is variable: it is most commonly observed in patients with nigrostriatal denervation, whereas cases with cerebellar or gait disturbance are usually unresponsive (83).

Considering that bilateral BG calcifications are common incidental findings, a substantial proportion of patients suffering from PD will have non-significant BG calcifications. Further workup is indicated in younger patients, if calcifications are not confined to pallidum, parkinsonism is not responsive to levodopa, and patients suffer from signs indicative of hypocalcaemia (paraesthesia, tetany and epileptic seizures), and/or disproportional neuropsychiatric symptoms.

#### 3.4.4 Osmotic demyelination syndrome

Osmotic demyelination syndrome occurs after rapid correction of hyponatraemia, but even gradual correction can produce myelinolysis in the setting of pre-existing malnourishment, alcoholism, Addison’s disease or immunosuppression. A biphasic course is typical. Initially, patients develop seizures or encephalopathy due to hyponatremia, but improve after electrolyte correction. Following a short period of stabilization (lasting 1–7 days), a second deterioration is caused by central nervous system demyelination. Central pontine myelinolysis (CPM) affects corticospinal and corticobulbar tracts, causing tetraparesis and pseudobulbar symptoms. Extrapontine myelinolysis (EPM) affects the midbrain, thalami and the BG and may cause parkinsonism, with prominent corticospinal signs due to co-existing CPM (85). Symptoms are symmetrical, but there are rare descriptions of asymmetric parkinsonism with dystonia and apraxia, mimicking corticobasal degeneration (86, 87). Parkinsonism may arise from demyelination in the SN, resulting in nigrostriatal denervation and abnormal functional dopaminergic imaging (88), or it may be the consequence of striatal myelinolysis (89). The response to levodopa depends on the relative damage to pre- and post-synaptic dopaminergic function. Parkinsonism in the setting of osmotic demyelination syndrome may be transient with full recovery or irreversible (90, 91). MRI demonstrates symmetric confluent T2/FLAIR hyperintensities and T1 hypointensities in the brainstem, striatum and thalamus (92, 93). Although pallidal sparing is considered typical for EPM, cases of isolated bipallidal involvement have been described (94). Importantly, there is an apparent lag between the onset of clinical features and appearance of MRI abnormalities on T2/FLAIR, with imaging changes lagging by up to 1–2 weeks. However, diffusion restriction can be seen as early as within 24 h of symptoms onset. In cases with high clinical suspicion but inconclusive early imaging, MRI should be repeated in 2–3 weeks.

### 3.5 Hypoxic-ischaemic brain injury

Hypoxic–ischemic brain injury (HIBI) is a consequence of compromised oxygen supply to the brain, which can result from decreased blood flow to the brain or hypoxia (95). The outcome depends on the mechanism of the hypoxic-ischaemic event, as these have distinct pathological consequences. Pure hypoxia does not necessarily lead to severe brain injury, even with prolonged lack of oxygen, as long as the systemic circulation is adequately preserved. Epidemiologically, these patients are younger, with less pre-existing atherosclerotic vascular disease, which has implications for cerebrovascular autoregulatory function (96, 97). A hypoxic event causes elevation of the partial pressure of carbon dioxide and respiratory acidosis, which triggers cerebrovascular dilation and an increase in cerebral blood flow. In the setting of a preserved systemic circulation, glucose supply to the brain continues, and toxic metabolites are washed away. Thus, after a purely hypoxic event, patients may be comatose, but have possibility for a full recovery. This is very different from the cardiac arrest in which the nutritional supply ceases and toxic metabolites accumulate. Hence, individuals who suffered significant HIBI may display different dynamics of neurological symptoms. Initially, patients have a diminished level of consciousness, which may lead to death or improvement, which can again be complete or partial. Moreover, apparent recovery may be followed by secondary deterioration after days to weeks (96, 97). Patients with HIBI who develop parkinsonism are older and with shorter latency of symptoms compared to those who develop a pure dystonic syndrome (97). The most common pathological correlate of parkinsonism caused by HIBI, both in animal models and in humans, is the necrosis of the pallidum and the striatum. CT shows low density lesions in these structures, while MRI shows T2-weighted/FLAIR hyperintense signals (97).

### 3.6 Intoxications

#### 3.6.1 Methanol poisoning

Outbreaks of methanol poisoning most commonly occur from illegal adulteration of ethanol with methanol. A massive poisoning took place recently in Iran during the COVID-19 pandemic, led by a belief that consumption of alcohol may prevent viral infection (98). Ingested methanol is metabolized in the liver to produce formaldehyde and formic acid, which causes metabolic acidosis and is also directly toxic for mitochondrial metabolism. Acute methanol intoxication results in coma, but with intensive treatment most patients survive, though parkinsonism may develop. Affected patients are blind due to methanol-induced optic neuropathy. The pathological correlate of methanol-induced parkinsonism is bilateral putaminal necrosis. CT shows bilateral putaminal hypodensities, sometimes with intermingling high density in the acute phase, suggestive of microhaemorrhage. MRI characteristics are time dependent. In the acute phase, there is low signal on T1-weighted imaging (but high if hemorrhages are present), high signal on T2/FLAIR scans and restricted diffusion on diffusion-weighted imaging due to cytotoxic oedema (99). In the chronic phase, bilateral biconvex lens-shaped putaminal T2/FLAIR hyperintense lesions with cavitations are characteristic (100). Some patients may respond to dopaminergic therapy, suggesting that methanol may affect the nigrostriatal pathway, while leaving some postsynaptic neurons intact to benefit from medications.

#### 3.6.2 Carbon monoxide poisoning

**Figure 2:**
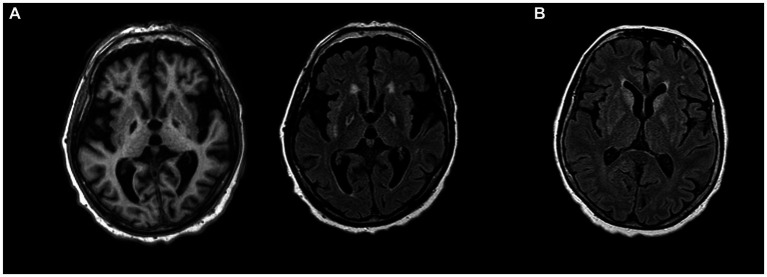
**(A)** T1-weighted hypointensities (left) and T2-weighted hyperintensities (right) bilaterally in globus pallidus in a patient with parkinsonism and cognitive decline after carbon monoxide posioning. **(B)** FLAIR hyperintensities in bilateral basal ganglia in anti-Ma2 encephalitis presenting with parkinsonism.

Toxic exposure to carbon monoxide (CO) may arise from motor vehicle exhausts, home heating furnaces, kitchen ovens, space heaters, and fires. CO blocks oxygen binding to hemoglobin and also directly inhibits mitochondrial cytochrome oxidase, further affecting the process of oxidative phosphorylation (101). The rate of reported neurologic sequels among survivors varies from 0.2–40%. Affected patients may show acute progressive or delayed relapsing course (102). In the acute form, victims do not regain consciousness, but progress from coma to the persistent vegetative state, remaining mute and bed-bound with spasticity, rigidity and paucity of spontaneous movements. Delayed sequels appear after consciousness is regained. These patients may look like they have recovered completely (to the extent that they are discharged home), but within a month abruptly develop parkinsonism and cognitive decline, sometimes with fixed limb dystonia. Symptoms may then progress to an akinetic-mute state or may stabilize and subsequently improve with minimal residual features (102). Dopaminergic drugs are ineffective, but spontaneous recovery occurs. The anatomical basis of CO induced parkinsonism is not straightforward, but likely results from bilateral pallidal lesions. CT reveals bilateral hypodense lesions in the GP and diffuse WM changes, but similar signal alterations are also observed in asymptomatic individuals. On MRI, lesions are hypointense on T1 and hyperintense on T2/FLAIR (Figure 2A). Diffusivity alterations in the acute phase may help identify patients at risk of developing parkinsonism (103).

#### 3.6.3 Cyanide poisoning

Acute cyanide poisoning is usually encountered in attempted suicide or homicide. Cyanide interferes with mitochondrial respiration by inhibiting cytochrome oxidase. Survival is uncommon and the primary long-term neurologic complication is parkinsonism, which may develop acutely or following a period of apparent recovery (104). The predominant neuropathological lesion involves the GP and SN pars reticularis, while the pars compacta remains intact (105). However, there are case reports with documented progressive loss of dopamine transporter suggestive of nigral neuronal apoptosis (106).

#### 3.6.4 Carbon disulfide and disulfiram intoxication

Carbon disulfide is a clear and colorless highly volatile liquid used in the viscose rayon and rubber industries, and as a fumigant for the sterilization of grain. The mechanism of its neurotoxicity is unknown (101). Neurological complications include parkinsonism. Primates chronically exposed to carbon disulfide develop a parkinsonism-like syndrome with extensive symmetrical necrosis of the GP and SN pars reticularis (107). Neurotoxicity of the drug disulfiram, used in the treatment of alcoholism, is likely related to its metabolite carbon disulfide. Parkinsonism may develop either in the setting of acute overdose or after days to weeks of disulfiram treatment (108). MRI shows lentiform lesions, affecting the pallidum and posterior putamen. Although disulfiram is almost no longer used in the treatment of alcoholism, it has been investigated as a repurposed drug for cancer treatment (109). Therefore, new cases of toxicity may appear in the future.

#### 3.6.5 Manganese intoxication

Manganese-induced parkinsonism has been reported in individuals with chronic occupational exposure to high Mn levels, among welders, miners or workers in dry battery manufacturing (101, 110). Mn intoxication has also been described in patients with chronic liver disease, on long-term parenteral nutrition (111, 112), and in Eastern European drug addicts following repetitive IV use of the recreational stimulant ephedrone (113). High Mn concentrations in mitochondria lead to oxidative stress. Recent reports suggest that Mn may affect misfolding of proteins such as α-synuclein and amyloid as well as play a role in neuroinflammation (114). Chronic manganism presents with parkinsonism, craniocervical and limb dystonia, and balance disturbances. Dystonic involvement of the legs is striking, resulting in the so-called ‘cock-gait’, with patients walking on their toes with their heels lifted off the ground (due to dystonic plantar flexors), with an erect spine and bent elbows (due to generalized dystonia) (115, 116). MRI is characteristic as it shows T1 hyperintensities in the pallidum. In the historical series of 13 Chilean miners with manganism, eight showed marked improvement with high levodopa doses (117). However, most subsequent papers reported levodopa unresponsiveness (118, 119). Levodopa should nevertheless be tried in all patients with Mn-induced parkinsonism.

#### 3.6.6 Drugs of abuse

Several illicit drugs have been associated with the development of parkinsonism. The tragic story behind 1-methyl-4-pyridine-1,2,3,6-tetrahydropyridine (MPTP) led to a major breakthrough in PD research. MPTP is a byproduct in the synthesis of a synthetic opioid, which causes selective degeneration of the SN. This led to development of animal models of PD. (120)

Heroin vapor inhalation (or used additives) can have a noxious effect of the BG, and has been rarely associated with parkinsonism. MRI can reveal leukoencephalopathy, as well as T1- and T2-weighted signal alterations in the basal ganglia. Levodopa responsiveness has been reported (121). Methamphetamines and synthetic cannabinoids can also induce parkinsonism, with similar MRI patterns of T2/FLAIR white matter hyperintensities and GPi or more distributed BG hyperintensities (122, 123). Organic solvent inhatalation is a common form of substance abuse in younger individuals. Chronic toluene abuse can lead to nigrostriatal damage resulting in parkinsonism with rest tremor. Ataxia and cognitive impairment are commonly observed. T2 hypointensities can be observed in deep gray matter nuclei, and extensive damage to the basal ganglia has been reported (124).

### 3.7 Infections

When parkinsonism develops in the acute or convalescent phase of a febrile illness, an infective etiology should be considered. Parkinsonism may be due to a direct effect of bacterial or viral infection on dopaminergic or BG function, or due to a parainfectious immune process.

#### 3.7.1 Viral encephalitis

Movement disorders are frequent and disabling consequences of Japanese B encephalitis, which is endemic in Southeast Asia and Western Pacific. Parkinsonism is noted early, as soon as consciousness is regained, and residual disability is common (125, 126). MRI findings include bilateral but asymmetric thalamic lesions. Focal lesions may also affect the BG, SN, cerebellum, pons and cerebral cortex (126, 127). Some patients with predominant lesions in the SN have good response to levodopa (128, 129). West Nile encephalitis can also cause SP. It is usually transient, but may persist in patients with severe encephalitis. Bilateral T2/FLAIR hyperintense lesions have been observed in various areas, including the basal ganglia, thalamus, medial temporal lobes, pons and SN (130, 131). HIV-related encephalopathy can lead to basal ganglia damage and to parkinsonism. Postural instability and gait difficulties are prominent, while rest tremor is absent. Levodopa response is variable. MRI can be normal but may reveal cortical atrophy as well as basal ganglia alterations (132, 133). Other viruses including SARS-CoV-2 may cause SP due to infectious or post-infectious encephalitis, but are typically associated with normal MRI or unspecific findings (132, 134).

#### 3.7.2 Mycoplasma pneumoniae

Parkinsonism in the context of *Mycoplasma pneumoniae* infection emerges as the respiratory infection resolves (135–138). It is caused by bilateral striatal necrosis: caudate and putamen are selectively affected and oedematous, followed by necrosis and consequent cavitations. On MRI, this pathological sequence corresponds to initial T2/FLAIR hyperintensity of the oedematous striatum, later replaced by progressive atrophy and cavitations that appear hypointense on T1, hypo-hyperintense on T2/ FLAIR and hypodense on CT. Although rare, *Mycoplasma pneumoniae* should be considered in children and young adults with preceding respiratory illness, who develop acute parkinsonism associated with radiological evidence of bilateral striatal necrosis.

#### 3.7.3 Neurocysticercosis

In a series of 590 consecutive patients with neurocysticercosis, parkinsonism was the most common movement disorder, affecting less than 3% of patients (139). Parkinsonism was bilateral (though sometimes asymmetric), with prominent axial features and of subacute onset. It was not associated with a particular localization of the cysts, but with widespread large lesions, causing distortions of brain structures and hydrocephalus. Hence, it was frequently accompanied by symptoms of intracranial hypertension, cognitive impairment, pyramidal features, ataxia and epileptic seizures. Neurocysticercosis should be considered in patients with parkinsonism who live or have traveled to endemic areas, who show atypical features and rapid progression.

#### 3.7.4 Parkinsonism related to opportunistic infections in immunocompromised patients

Parkinsonism may be the initial manifestation of *Toxoplasma gondii* infection, with MRI showing ring-enhancing lesions in the BG (140). In a case of disseminated cerebral toxoplasmosis, presynaptic dopaminergic deficit and a positive response to levodopa were observed (141). Subacute parkinsonism in immunocompromised patients should also alert the physician to the possibility of cryptococcal meningoencephalitis (rarely seen also in immunocompetent patients) (142, 143) or HHV-6 (144). Acute/subacute parkinsonism (and other movement disorders) may also develop due to a toxic effect of immunosuppressive therapy (145).

#### 3.7.5 Parkinsonism in Creutzfeldt-Jakob disease

Parkinsonism may be a manifestation in the transmissible group of prion diseases. Akinetic mutism is common in the terminal stages of Creutzfeldt-Jakob disease (CJD). However, atypical parkinsonism may also be the presenting feature of variant or sporadic CJD (146, 147). The rapidly progressive course along with cognitive decline, myoclonus as well as other extrapyramidal and/or psychiatric features is suggestive of CJD (148). MRI reveals hyperintensities on diffusion-weighted and T2/FLAIR sequences. Sporadic CJD is characterized by cortical ribboning as well as alterations in deep gray matter nuclei (149). In variant CJD, MRI may reveal the pulvinar sign (bilateral hyperintensities in the pulvinar thalamic nuclei) or the hockey stick sign (additional involvement of the medial thalamus) (150).

### 3.8 Immunological causes

#### 3.8.1 Paraneoplastic and autoimmune parkinsonism

Paraneoplastic neurological syndromes are mostly immune-mediated disorders that occur in patients with cancer. They can affect any part of the nervous system. They are a consequence of an immune response initiated by onconeural antigens. They often precede other manifestations of cancer (151). Paraneoplastic parkinsonism has been described in the association with CRMP5, Ri, and Ma2-antibodies (152–156). A rapidly progressive and disabling course is characteristic, but insidious course is also possible (157). SP can also be associated with antibodies against neuronal surface antigens including antibodies against NMDA receptors, LGI1, CASPR2, and IgLON5. These disorders may commonly manifest without an underlying neoplasm (158).

Parkinsonism associated with Ma2 antibodies typically affects young men with testicular germ-cell cancer (154). Because of the characteristic vertical gaze palsy, they may phenotypically resemble PSP (154, 159). Additional movement disorders include masticatory dyskinesias, such as forceful jaw opening and closing, resulting in lip and tongue injuries. Due to involvement of limbic regions, diencephalon and upper brainstem, additional distinctive features may include memory impairment and signs of hypothalamic–pituitary dysfunction: weight gain, excessive daytime sleepiness, narcolepsy and cataplexy (160). In patients older than 50 years and in women, the underlying tumor is non-small-cell lung carcinoma, breast cancer, colon cancer, or lymphoma. Recognition of anti-Ma2 encephalitis is important, because one third of patients partially respond to tumor treatment and immunotherapy. The typical MRI patterns of Ma2-antibody encephalitis are FLAIR/T2 hyperintense signals in the medial temporal lobes, hypothalamus, thalamus, basal ganglia and upper brainstem, often with contrast enhancement (Figure 2B) (154). A rare but distinct phenotype has been described in young male patients with testicular tumor, consisting of extreme bradykinesia, severe rigidity, ocular motor and lid apraxia and reduced verbal output. In these cases, MRI always shows lesions affecting the SN, pallidum or both (154).

Parkinsonism associated with anti-Ri antibodies affects female patients with breast cancer (152). It may be an early manifestation, but can also develop later in the course of the disease. It is associated with supranuclear gaze palsy and/or cerebellar ataxia. The clinical course if often slower than in other paraneoplastic syndromes, mimicking neurodegenerative conditions such as PSP or mulitple system atrophy. MRI is often normal, but brainstem T2/FLAIR hyperintensive changes, including the SN, palllidum, caudate and putamen have been described.

Paraneoplastic parkinsonism associated with CRMP5 antibodies may also mimic PSP due to co-existing gaze palsy. Bilateral T2 hyperintensity of the BG is characteristic (155).

SP may appear in cases of autoimmune encephalitis associated with antibodies against neural surface antigens. Anti-NMDAR encephalitis is the most common autoantibody mediated encephalitis. It has prominent cognitive and psychiatric manifestations. Parkinsonism is also a possible manifestation of anti-NMDAR encephalitis, most commonly observed in children (158, 161). Interestingly, a case of worsening of parkinsonism in a patient with pre-existing PD due to anti-NMDAR encephalitis has also been described (162). Combined with other signs of encephalopathy and/or characteristic faciobrachial dystonic seizures, parkinsonism may also appear in anti-LGI1 encephalitis (158, 163). Parkinsonism has been observed in cases of anti-CASPR2 encephalitis, along with its more typical clinical features that include ataxia, seizures, neuromyotonia, autonomic dysregulation, mood changes and hyponatremia (164). In causes of autoimmune encephalitis, MRI findings are variable. Signal abnormalities are most commonly observed in the medial temporal lobes or the basal ganglia, but normal MRI is common and therefore does not rule out the diagnosis (165, 166). As these disorders are curable, early diagnosis and immunotherapy are crucial.

Anti-IgLON5 disease typically has a more insidious course, but may also progress subacutely. Parkinsonism is commonly associated with supranuclear gaze palsy (mimicking progressive supranuclear palsy) and gait instability. Other movement disorders, additional oculomotor abnormalities, bulbar symptoms, sleep and breathing disorder are also part of the clinical picture (158, 167). MRI is usually unremarkable. However, cases with atrophy of the hippocampus, brainstem and cerebellum have been described, as well as examples of restricted diffusion, hyperintense T2/FLAIR signal, hypointense T1 signal and/or gadolinium contrast enhancement in the brainstem, cerebellum and the cerebral hemispheres. It is likely that these different findings relate to the speed of clinical progression (168).

#### 3.8.2 Systemic autoimmune diseases

Parkinsonism is a rare manifestation of systemic autoimmune disorders. It has been reported in approximately 40 cases of systemic lupus erythematosus (SLE), a majority were young women with an already established diagnosis of SLE (169). Only rarely was subacute parkinsonism the first manifestation of SLE (170–172). MRI findings were non-specific and included WM changes, ventricular dilatation, cortical atrophy and infarcts, and were more pronounced in patients with co-existing antiphospholipid-antibodies (up to 75%) (169). Dopaminergic therapy was ineffective, but patients responded to corticosteroids and other immunosuppressive agents. There have been only 9 reported cases of parkinsonism in antiphospholipid syndrome (169), predominantly males in their fifties. The mechanism was thrombo-occlusive vasculopathy, leading to irreversible neuronal loss evident on MRI as infarctions within the BG. Dopaminergic therapy was ineffective. Parkinsonism is rarely reported in Sjogren’s syndrome, predominantly in women with mean age of onset of 60 years. It is thought to be caused by an underlying vasculitis. MRI shows T2/FLAIR hyperintensities in the WM, in the striatum or in the pallidum. Dopaminergic therapy is ineffective, but both parkinsonism and MRI changes respond to corticosteroids (169, 173).

#### 3.8.3 Multiple sclerosis

There has been a number of reported cases of parkinsonism in patients with multiple sclerosis. Often, a chance rather than causal association was the more likely explanation, because neither a correlation with MRI lesions in expected locations nor a response of parkinsonism to corticosteroids could be established. Moreover, there are reports where a clear genetic cause of PD was demonstrated in patients with parkinsonism and co-existing multiple sclerosis, further confirming co-occurrence by chance (174, 175). Nevertheless, there are cases where central demyelinating lesions in the bilateral thalamus and pallidum or SN could be closely linked to symptoms of parkinsonism, with clear response to corticosteroids (176).

### 3.9 Miscellaneous causes

There have been few reports of parkinsonism after wasp sting, associated with bilateral striatal and pallidal necrosis (177, 178). An immune-mediated mechanism was suggested. Parkinsonism was rarely associated with ethylene glycol toxicity, with MRI showing haemorrhagic necrosis of the thalamus and lentiform nuclei (179). While non-ketotic hyperglycemia is classically associated with acute hemichorea/hemibalismus, parkinsonism may occasionally emerge after remission of chorea, with MRI showing regression of T1 hyperintensities (characteristic for the acute stage with chorea), with persisting striatal atrophy and T2 hyperintensities in the parkinsonian phase (180). There is a single case report of reversible parkinsonism after a hypoglycaemic episode, with bilateral BG T2/FLAIR lesions presumably caused by vasogenic oedema (181).

## 4 Discussion

### 4.1 Relevance of secondary parkinsonism for clinical practice

Although rare compared to PD, SP should always be considered in the differential diagnosis, since it has a different prognosis. The localization and imaging characteristics of the lesions may lead to identification of the specific cause, which allows for specific treatment.

### 4.2 Relevance of secondary parkinsonism for understanding Parkinson’s disease

Secondary causes of parkinsonism have contributed to the knowledge on the pathophysiology and treatment of PD. Based on the pathological finding of a tuberculoma destroying the SN in a patient with contralateral parkinsonism, Edouard Brissaud put forward the hypothesis that the SN is the major site of pathology in PD. (182) Later recognition that lesions outside the nigrostriatal circuits may cause parkinsonism led to the understanding of parkinsonism as a network disorder (183). The identification of MPTP toxin, and further development of the primate model of PD, had a significant impact on the understanding of PD pathophysiology and led to the identification of the subthalamic nucleus as a target for deep brain stimulation (120). The selective vulnerability of the BG to mitochondrial toxins such as cyanide or carbon monoxide stands as a proof of the high dependence of BG neurons on energy production and their susceptibility to excitotoxic and oxidative stress, which resonates with mechanisms of selective vulnerability of the BG in the neurodegenerative forms of movement disorders.

### 4.3 Anatomical considerations, network impact of the lesions and relevance for clinical neuroscience

SP has been associated with lesions at different brain locations, most closely to the lesions in the mesencephalon, which cause nigrostriatal dopaminergic deficit, resulting in parkinsonism that closely resembles idiopathic PD (9, 184). Lesions of the BG may also produce parkinsonism, although other movement disorders are more common, particularly dystonia (185). Parkinsonism has most commonly been observed in bilateral lentiform lesions, but only in a minority of cases with such lesions, underscoring the importance of individual factors in the vulnerability of BG circuits. Specific imaging abnormalities in patients with SP due to metabolic or toxic causes indicate a central role of pallidum, but again only a proportion of patients with pallidal lesions develop parkinsonism (102, 186). Finally, in contrast to what could be expected based on the established view of the functional organization of the BG, vascular damage to the thalamus has not been linked to post-stroke parkinsonism (187–189). Interestingly, thalamic strokes have been associated with disappearance of PD resting tremor (190). Hence, correlations between the site of the lesion and the emergence of signs of parkinsonism are equivocal.

Modern analytical methods better account for the varied clinical presentations of patients with BG lesions by taking into account similar symptoms arising from lesions in different locations and remote functional alterations in connected but intact brain regions (191, 192). In a study of imaging and clinical data of 29 patients with parkinsonism and localized brain damage due to various causes and in different areas, a common network of brain regions associated with parkinsonism was identified. Over 90% of lesion locations were connected to the midbrain, BG, the anterior cingulate cortex and the cerebellum. However, connectivity to the claustrum was most sensitive and specific for lesion-induced parkinsonism, suggesting the claustrum as a potential novel therapeutic target (183). Using an analogous approach, lesions in areas functionally connected to the dorsal medial cerebellum were associated with freezing of gait (193).

## 5 Conclusion

SP associated with structural brain changes is an important clinical entity that has also helped to understand parkinsonism as a network disorder. Compared to other movement disorders associated with brain lesions, parkinsonism is infrequent, suggesting the strong resilience of the parkinsonian network that may be due to a pre-existing reserve in dopaminergic function and/or due to strong adaptive capacity of the BG-thalamo-cortical and the cerebello-thalamo-cortical networks. Recognition that, in the setting of a specific cause, only a proportion of cases with identifiable BG lesions develop parkinsonism (or other movement disorders) also stresses the importance of individual vulnerability in health and disease. Finally, toxic causes of SP inform us on the selective susceptibility of the BG to the energy failure from mitochondrial dysfunction, the pathophysiological mechanism also applicable to much more common forms of neurodegenerative parkinsonism.
